# Prognostic performance of preoperative systemic inflammatory biomarkers in upper tract urothelial carcinoma after radical nephroureterectomy: a systematic review and meta-analysis of NLR, MLR, PLR, SII and SIRI

**DOI:** 10.3389/fonc.2026.1899751

**Published:** 2026-07-29

**Authors:** Guopeng Wang, Xiang Dong, Xintong Zhang, Chengpeng Gu, Ting Hu, Fei Yang, Dong Wang, Yu Mao, Shangqing Ren

**Affiliations:** 1 School of Medicine, University of Electronic Science and Technology of China, Chengdu, China; 2 Robotic Minimally Invasive Center, Sichuan Provincial People’s Hospital, Chengdu, Sichuan, China

**Keywords:** biomarkers, inflammation, meta-analysis, nephroureterectomy, prognosis, urothelial carcinoma

## Abstract

**Background:**

Risk stratification remains challenging in patients with upper tract urothelial carcinoma (UTUC) undergoing radical nephroureterectomy (RNU). Preoperative systemic inflammatory biomarkers may provide prognostic information, but their relative clinical utility remains unclear.

**Methods:**

This systematic review and meta-analysis evaluated five commonly used preoperative inflammatory biomarkers—neutrophil-to-lymphocyte ratio (NLR), platelet-to-lymphocyte ratio (PLR), monocyte-to-lymphocyte ratio (MLR), systemic immune-inflammation index (SII), and systemic inflammation response index (SIRI)—in patients with UTUC treated with RNU. PubMed, Embase, Web of Science, and Cochrane Library were searched up to March 2026. Multivariate hazard ratios (HRs) for overall survival (OS), cancer-specific survival (CSS), and recurrence-free survival (RFS) were pooled using random-effects models. Evidence certainty was assessed using the Grading of Recommendations Assessment, Development and Evaluation (GRADE) approach.

**Results:**

Twelve retrospective studies comprising 7,346 patients were included. Elevated inflammatory biomarker levels were generally associated with worse survival outcomes across available studies. Significant associations with overall survival were observed for SIRI (pooled HR 2.09, 95% CI 1.27–3.44), NLR (pooled HR 1.97, 95% CI 1.74–2.22, I²=14.1%), MLR, SII, and PLR; comparable associations were seen for cancer-specific and recurrence-free survival. Each biomarker was analysed in a separate meta-analysis, and the contributing studies differed in design, populations, and cut-off values; the markers were therefore not compared statistically, and no inference regarding their relative prognostic performance is made.

**Conclusion:**

Preoperative systemic inflammatory biomarkers are individually associated with oncological outcomes following RNU for UTUC. Because each marker was pooled separately and the studies were clinically heterogeneous, the markers were not compared statistically and no ranking is implied; the findings are descriptive and hypothesis-generating. This meta-analysis of aggregate data did not assess predictive performance (discrimination, calibration, or clinical utility), and any SIRI–NLR combination remains a hypothesis requiring prospective, individual-patient-data validation before any clinical application.

**Systematic review registration:**

https://www.crd.york.ac.uk/PROSPERO/view/CRD420261307425, identifier CRD420261307425.

## Introduction

Upper tract urothelial carcinoma (UTUC) is a rare urological malignancy accounting for approximately 5–10% of all urothelial carcinomas, with an increasing incidence in recent decades. Radical nephroureterectomy (RNU) with bladder cuff resection is the standard treatment for muscle-invasive or high-risk non-muscle-invasive UTUC (1). Despite standardised surgical management, oncological outcomes remain heterogeneous and vary substantially according to tumour stage, grade, nodal status, and other clinicopathological factors. Recurrence and cancer-specific mortality remain clinically important concerns after RNU. Currently, clinicopathological parameters (e.g., T stage, grade, lymph node status) are the main prognostic tools for UTUC, but they fail to fully capture the biological heterogeneity of the disease. Tumour-promoting inflammation is now recognised as an enabling characteristic of cancer, fostering proliferation, angiogenesis, immune evasion, and metastasis, and the systemic inflammatory response is partly reflected in circulating blood-cell populations (2, 3). Thus, identifying reliable, low-cost, and easily accessible prognostic biomarkers is critical to optimise risk stratification and guide personalised postoperative surveillance and adjuvant therapy.

NLR, PLR, MLR, SII, and SIRI are the most frequently reported peripheral-blood inflammatory indices in UTUC and capture complementary aspects of the systemic inflammatory response, from single leucocyte ratios to composite indices incorporating platelet and monocyte counts. However, previous meta-analyses have each examined a single marker in isolation (4, 5), so the evidence for these markers has not been consolidated within a single UTUC/RNU population. Therefore, this study aimed to summarise, descriptively, the prognostic association of each of these five preoperative inflammatory biomarkers with comparable survival endpoints. The aim was not to introduce a new biomarker—each of these five indices has already been studied individually in UTUC—but to consolidate the existing evidence for all five within one comparable UTUC/RNU population, so that their pooled effect magnitudes and the robustness of that evidence can be viewed side by side. The intention is to provide an evidence baseline and to identify priorities for future prospective, comparative studies, rather than to establish the statistical superiority of any single marker.

## Materials and methods

### Search strategy and study selection

The protocol of this systematic review and meta-analysis was registered in PROSPERO (CRD420261307425) and conducted in accordance with PRISMA 2020 recommendations (6). A comprehensive literature search was conducted in PubMed, Embase, Web of Science, and Cochrane Library up to March 2026 (detailed search strings in **Supplementary Table 1**). Reference lists of relevant articles were manually screened. Two independent reviewers screened titles and abstracts and assessed full texts for eligibility (κ = 0.92; **Supplementary Table 2**). A total of 532 records were identified. The review question was structured according to a PECO framework: patients with UTUC undergoing RNU; exposure to elevated preoperative systemic inflammatory biomarkers; comparison with low biomarker levels; and oncological outcomes including OS, CSS, and RFS.

Several amendments were made to the registered protocol before data analysis began, consistent with the PROSPERO revision history. First, the pre-registered network meta-analysis was not feasible because of an insufficiently connected evidence network and limited overlapping data across biomarkers, and was therefore replaced by separate random-effects meta-analyses for each biomarker. Second, the systemic inflammation response index (SIRI) replaced the fibrinogen-to-albumin ratio (FAR) as the fifth biomarker, because FAR was reported by too few studies (fewer than three) whereas SIRI was adequately reported. Third, risk of bias was assessed using the Newcastle-Ottawa Scale rather than the pre-registered Cochrane risk-of-bias tool, because all included studies were retrospective observational cohorts, for which tools designed for randomised trials are not applicable. Fourth, the pre-specified assessment of publication bias was not performed because every biomarker–endpoint combination included fewer than 10 studies (see Statistical analysis).

### Inclusion and exclusion criteria

Studies were included if they (i) investigated UTUC patients treated with RNU and (ii) reported multivariate HRs with 95% CIs for at least one of the five markers on OS, CSS, or RFS. Studies were excluded if they (i) lacked sufficient data to extract HRs and 95% CIs; (ii) were conference abstracts, case reports, reviews, or basic research articles; (iii) had incomplete or overlapping study populations with other included studies; (iv) did not focus on the prognostic role of inflammatory biomarkers in UTUC; or (v) had a sample size of fewer than 50 patients.

### Data extraction and quality assessment

Two reviewers independently extracted study characteristics, HRs, and cut-off values. Study quality was assessed using the Newcastle-Ottawa Scale (NOS; **Table 1**; **Supplementary Figure 1**) (7). The NOS was selected because all included studies were retrospective observational cohorts and the NOS is widely applied to such designs in prognostic meta-analyses, providing transparent and comparable domains for selection, comparability, and outcome assessment. We acknowledge that the Quality In Prognosis Studies (QUIPS) tool has been specifically developed for prognostic-factor reviews and is increasingly recommended; the implications of this choice are considered in the Limitations.

**Table 1:** Baseline characteristics and quality assessment of included studies.

Study ID	Year	First author	Country	n	Main biomarkers	Outcomes	NOS score
Boubaker_2025	2025	Boubaker NS	Tunisia	107	MLR, NLR, PLR, SII, SIRI, combinations	OS, Local RFS	8
Jan_2019	2019	Jan HC	Taiwan	424	MLR, SII	OS, CSS	8
Chien_2021	2021	Chien TM	Taiwan	376	NLR, PLR, SII	CSS, MFS, BRFS	8
Ghorai_2024	2024	Ghorai RP	India	91	NLR	OS	7
Tan_2018	2018	Tan P	China	717	NLR	OS, CSS, RFS, MFS	8
Son_2018	2018	Son S	Korea	1137	NLR, PLR	RFS, CSS	7
Tanaka_2014	2014	Tanaka N	International	665	NLR	RFS, CSS	9
Dalpiaz_2017	2017	Dalpiaz O	Europe	180	PLR	OS, CSS	8
Zheng_2019	2019	Zheng Y	China	259	PLR, SIRI, SIRI+PLR	OS, CSS, MFS (train)	8
Zheng_2020	2020	Zheng Y	China	253	SII	OS, CSS, RFS (train)	9
Mori_2021	2021	Mori K	International	2492	SII	OS, CSS, RFS	9
Yu_2024	2024	Yu LC	China	645	SIRI	OS, CSS, RFS	8

Total patients = 7,346. Only training-cohort data were extracted from Zheng_2019 and Zheng_2020 to avoid duplication bias. NLR, neutrophil-to-lymphocyte ratio; PLR, platelet-to-lymphocyte ratio; MLR, monocyte-to-lymphocyte ratio; SII, systemic immune-inflammation index; SIRI, systemic inflammation response index; OS, overall survival; CSS, cancer-specific survival; RFS, recurrence-free survival; MFS, metastasis-free survival; BRFS, bladder-recurrence-free survival; NOS, Newcastle-Ottawa Scale.

### Statistical analysis

As noted above, a formal network meta-analysis was not feasible because of the lack of a connected evidence network and insufficient direct comparisons across all biomarkers. Each biomarker was therefore analysed in a separate meta-analysis, and no formal direct or network-based comparative estimates between biomarkers were derived. Random-effects meta-analysis (inverse-variance method, DerSimonian-Laird estimator) was performed (8). Heterogeneity was quantified by I² (9). Subgroup analyses (region, sample size, NOS score) were conducted for SIRI. Leave-one-out sensitivity analysis was performed for SII. Formal assessment of publication bias (funnel-plot asymmetry, Egger’s test, and trim-and-fill adjustment) was not performed because every biomarker–endpoint combination included fewer than 10 studies, below the threshold at which such tests are reliable (10). Sources of heterogeneity were explored using Baujat plots. Evidence certainty was assessed using the Grading of Recommendations Assessment, Development and Evaluation (GRADE) approach and rated as low to very low (11), mainly due to the retrospective design of all studies and inconsistency in cut-off values. Statistical analyses were conducted in R (version 4.5.3). The certainty of evidence is presented in **Supplementary Table 3**.

## Results

### Study selection

**Figure 1:**
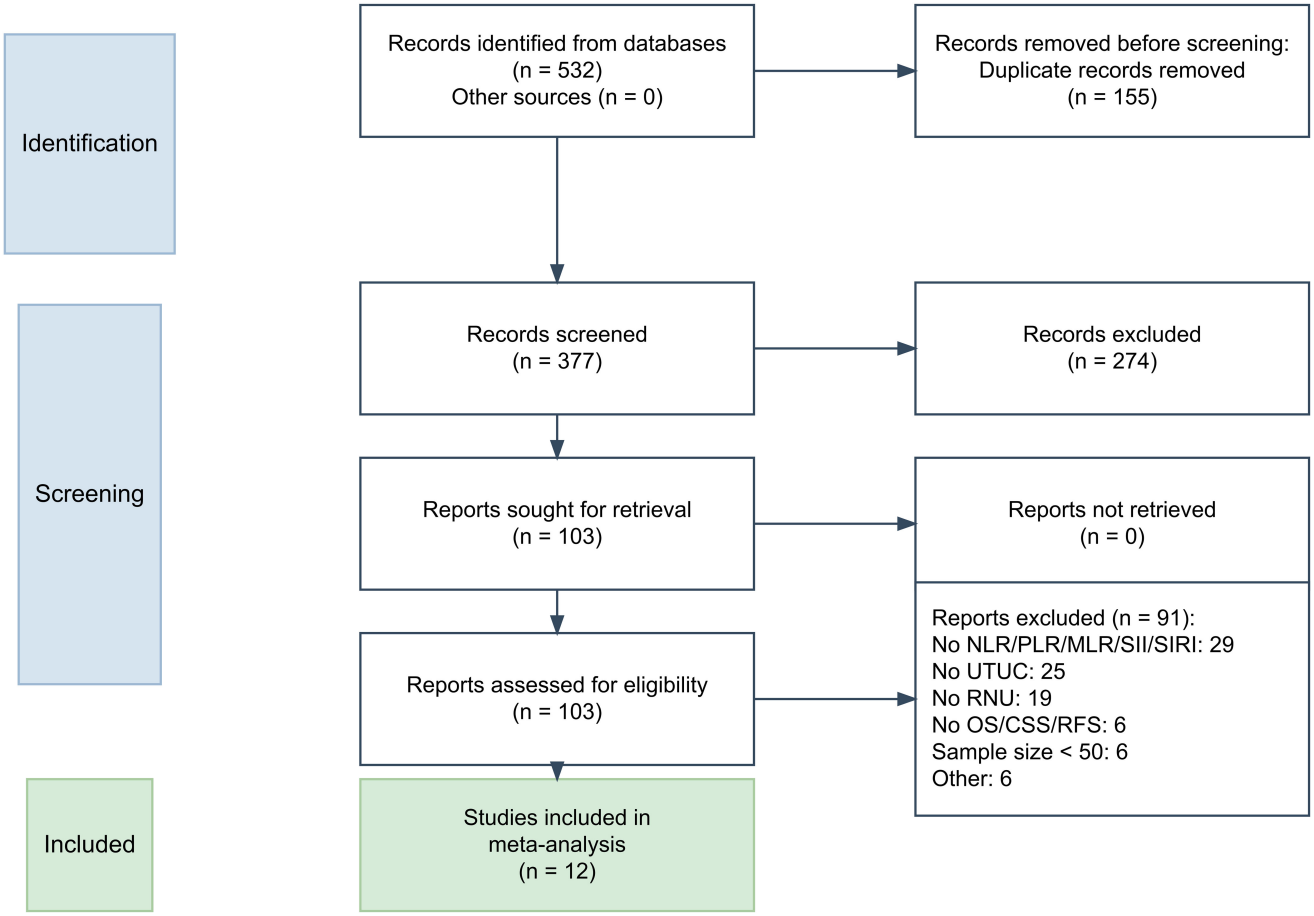
PRISMA 2020 flow diagram of study selection for this systematic review and meta-analysis.

After removal of duplicates and two-stage screening, 12 unique studies involving 7,346 patients were included (**Figure 1**) (12–23).

### Study characteristics

Baseline characteristics, quality assessment (NOS), and biomarker cut-off values are summarised in **Table 1**, **Supplementary Figure 1**, and **Supplementary Table 4**. Most outcomes were rated as low to very low, primarily due to the retrospective design of all studies, inconsistency in cut-off values, and the limited number of studies for some markers.

### Meta-analysis

**Figure 2:**
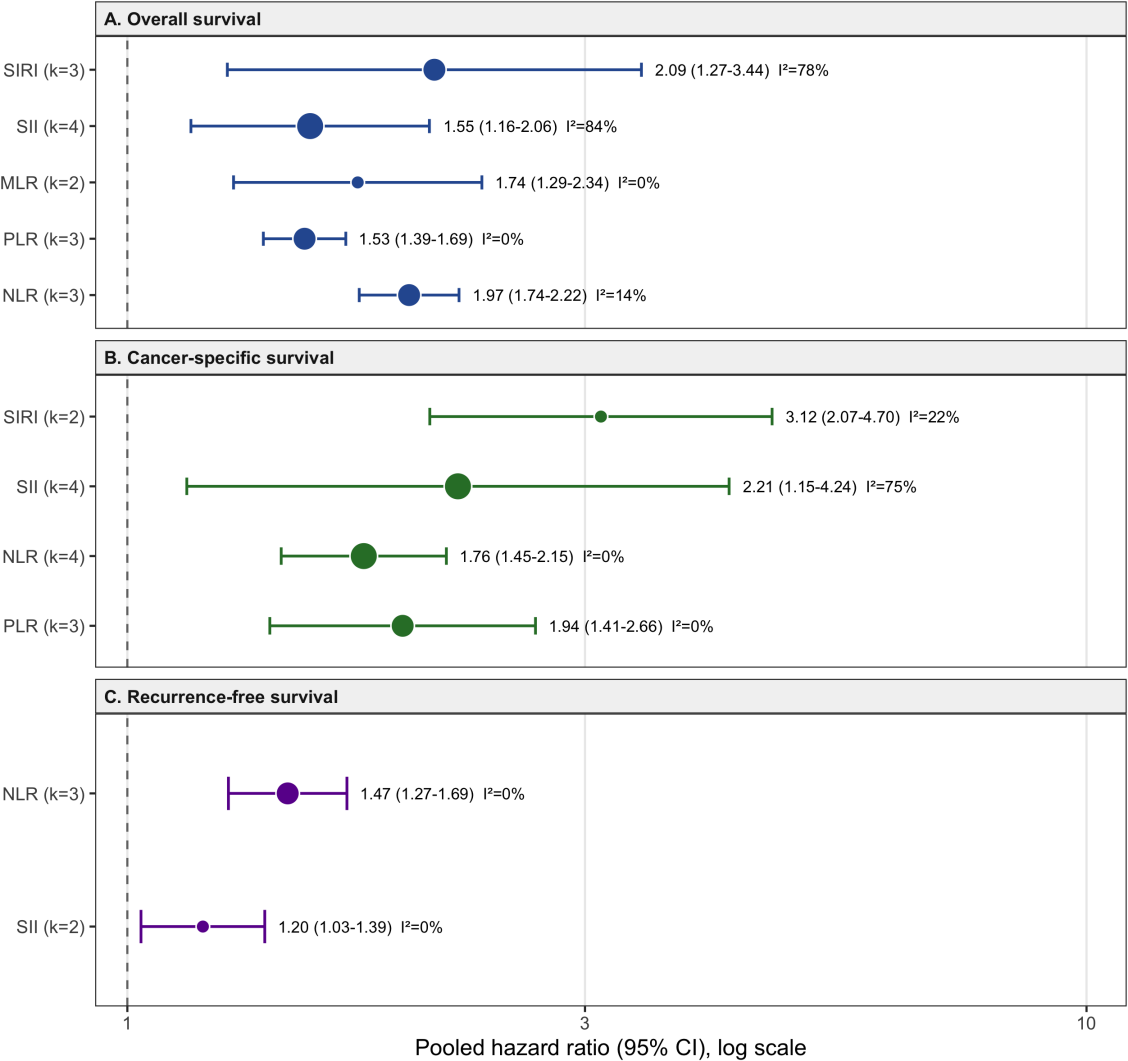
Forest plots of pooled hazard ratios (HRs) for **(A)** overall survival (OS), **(B)** cancer-specific survival (CSS), and **(C)** recurrence-free survival (RFS) according to preoperative inflammatory biomarkers.

**Figure 3:**
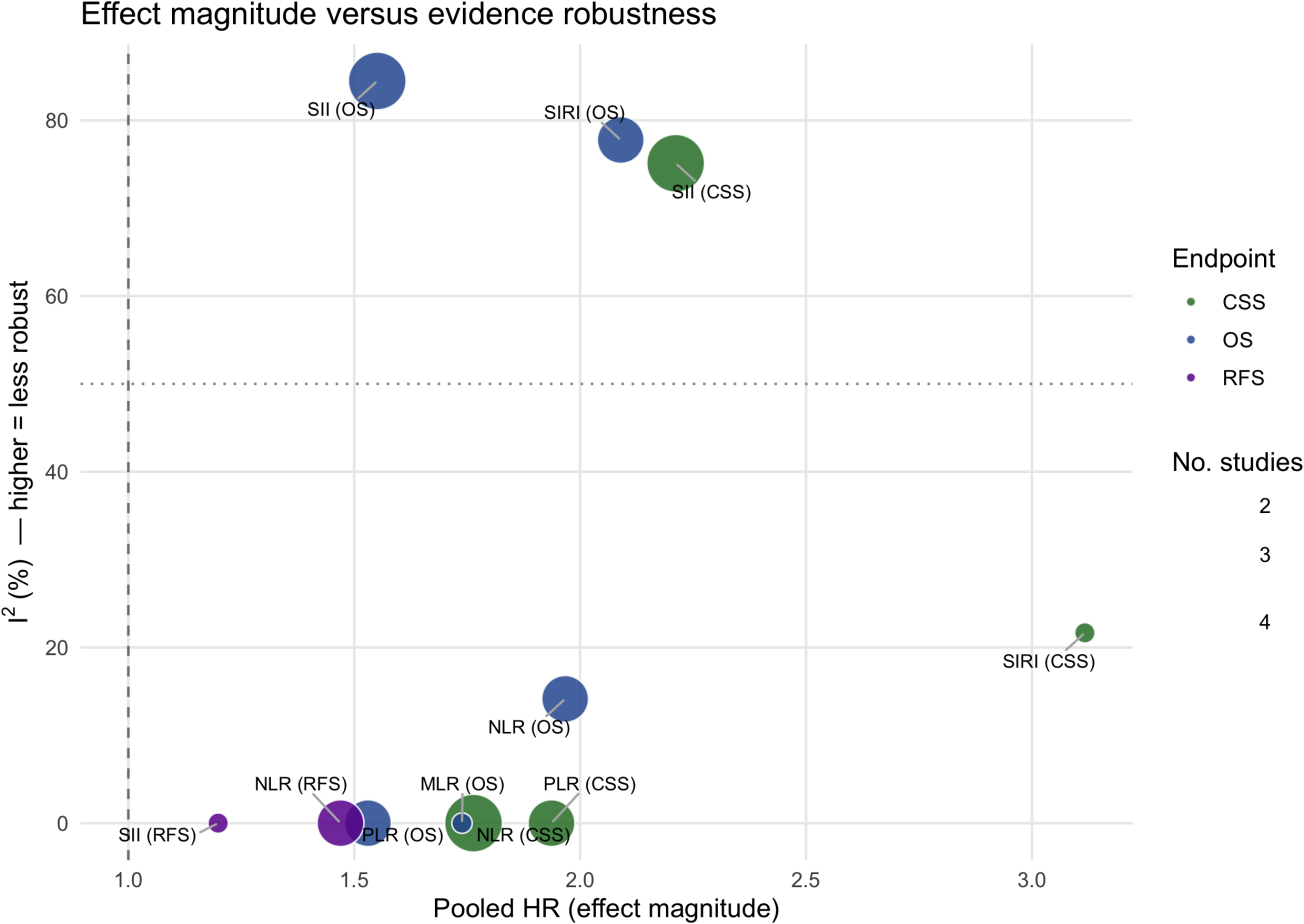
Comparative summary of the five inflammatory biomarkers across endpoints, plotting pooled effect magnitude (hazard ratio, x-axis) against statistical heterogeneity (I², y-axis); bubble size is proportional to the number of pooled studies and colour denotes the endpoint (OS, CSS, RFS). This figure is intended for descriptive visualisation only. Because each biomarker was pooled in a separate meta-analysis, and the contributing studies differed in populations and cut-off values, the plot does not represent a formal statistical comparison or ranking of biomarkers, and the relative positions should not be interpreted as evidence of the superiority of one marker over another.

**Figure 4:**
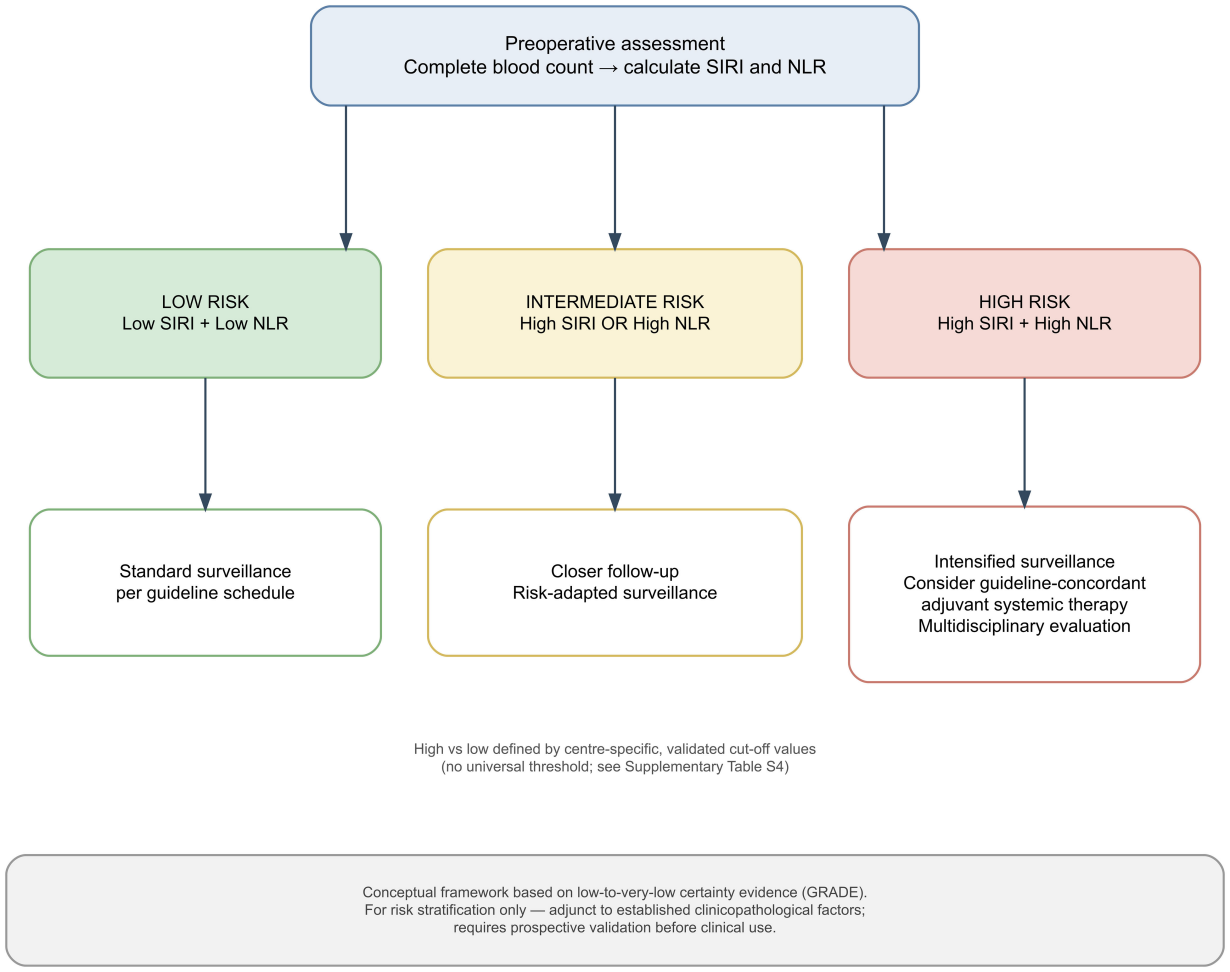
Entirely conceptual and hypothesis-generating schematic illustrating how preoperative SIRI and NLR, both derived from routine complete blood counts, might in principle be considered together. This figure is provided for illustration only and is not a validated risk-stratification tool: no included study evaluated a combined SIRI–NLR model, and the review did not assess the discrimination, calibration, or clinical utility of any such scheme. The grouping shown (low, intermediate, high) is a conceptual hypothesis to be tested in future prospective, individual-patient-data studies and must not be used to guide current clinical management. Any cut-off values would need to be defined and validated externally (see **Supplementary Table 4**). The framework is based on low-to-very-low certainty evidence (GRADE).

Across the three endpoints, each biomarker could be pooled from two to four studies, and elevated levels were associated with worse outcomes in all significant analyses (**Figures 2**, **3**). For overall survival (OS), significant associations were observed for SIRI (pooled HR 2.09, 95% CI 1.27–3.44, I²=77.8%), NLR (pooled HR 1.97, 95% CI 1.74–2.22, I²=14.1%), MLR, SII, and PLR (**Figure 2A**). For cancer-specific survival (CSS), significant associations were observed for SIRI (HR 3.12, 95% CI 2.07–4.70), SII (HR 2.21, 95% CI 1.15–4.24, I²=75.1%), PLR (HR 1.94, 95% CI 1.41–2.66), and NLR (HR 1.76, 95% CI 1.45–2.15), the last two with no heterogeneity (I²=0%) (**Figure 2B**). For recurrence-free survival (RFS), NLR (pooled HR 1.47, 95% CI 1.27–1.69, I²=0%) and SII (HR 1.20, 95% CI 1.03–1.39, I²=0%) were significantly associated with recurrence (**Figure 2C**); SIRI and PLR were each reported in only a single RFS study and were therefore not pooled. Each biomarker was pooled in a separate meta-analysis, and the contributing studies differed substantially in design, populations, cut-off definitions, and adjustment strategies; the markers were therefore not compared head-to-head within a unified statistical model, and no inference regarding the relative superiority of one biomarker over another is made. Accordingly, **Figure 3** is presented for descriptive visualisation only and does not represent a formal statistical comparison or ranking of biomarkers. The corresponding forest plots for each individual biomarker–endpoint analysis are provided in **Supplementary Figure 2**. A conceptual framework based on these observations is presented in **Figure 4** and discussed, with its limitations, below.

### Subgroup and sensitivity analyses

Exploratory subgroup analyses for SIRI were performed according to study sample size, geographic region, and NOS score. The prognostic association of SIRI with poor outcomes remained consistent across these subgroups (**Supplementary Figure 3**). Due to the limited number of studies (n=3), formal tests for interaction were not powered and should be interpreted with caution. For SII (I²=84.5% for OS), leave-one-out sensitivity analysis identified the large international multicentre study by Mori et al. (2021) as the primary source of heterogeneity (**Supplementary Table 5**; **Supplementary Figure 4**) (20). This study enrolled European and American populations, and its cut-off values differed from those used in the predominantly Asian cohorts, which may partly explain the substantial heterogeneity observed for SII. This finding was further supported by the Baujat plot (**Supplementary Figure 5**). However, because these explanations were based on study-level characteristics rather than patient-level data, they should be considered hypothesis-generating.

## Discussion

This study summarises, for the same UTUC/RNU setting, the pooled association of each of five inflammatory markers with survival, together with the heterogeneity and robustness of the underlying evidence. It is intended as a descriptive synthesis of prognostic associations, not as a statistical comparison or ranking of biomarkers: each marker was analysed in a separate meta-analysis, and the contributing studies differed substantially in design, populations, cut-off definitions, and adjustment strategies, so no inference regarding relative biomarker superiority is warranted. Each individual pooled estimate is consistent with earlier meta-analyses confined to a single index (for example, pooled OS hazard ratios of approximately 1.64 for NLR (4) and 1.87 for SII (5)), supporting the validity of the present pooled results. For SII, leave-one-out sensitivity analysis identified the large multicentre study by Mori et al. (2021) as the main contributor to heterogeneity (omission reduced I² from 84.5% to 0%; **Supplementary Table 5**; **Supplementary Figures 4**, **5**); for NLR, excluding the statistical outlier Ghorai et al. (2024) from the OS analysis left the pooled estimate essentially unchanged while reducing I² to 0% (**Supplementary Table 6**). A formal network meta-analysis, which would be required for any direct comparative inference, was not feasible because of the limited overlapping data across biomarkers; the descriptive observations presented here should therefore be regarded as exploratory and hypothesis-generating only.

### Biological rationale for SIRI

Although based on limited evidence, SIRI was originally developed as an integrated index calculated from peripheral neutrophil, monocyte, and lymphocyte counts (24). In the present context, SIRI integrates neutrophils and monocytes in the numerator while using lymphocytes in the denominator, thereby capturing both the pro-tumour inflammatory drive and the monocyte/macrophage axis. This dual-component design provides a more comprehensive reflection of the systemic inflammatory and immunosuppressive tumour microenvironment than single-ratio markers (25).

### Clinical implications and decision-making framework

These biomarkers are prognostic associations rather than validated predictive tools. No included study evaluated a combined SIRI–NLR model, and no analysis in this review assessed discrimination (e.g., C-index or AUC), calibration, or clinical utility (e.g., decision-curve analysis), nor whether the joint use of the two markers adds prognostic value beyond either marker alone or beyond established clinicopathological factors such as tumour stage, grade, lymphovascular invasion, or surgical margin status. Such analyses require individual patient data and a formal predictive-modelling design, and could not be performed from the published aggregate hazard ratios available for this review. Any dual-marker strategy is therefore entirely hypothesis-generating, and the present data do not support its use to guide clinical decisions. The available evidence is insufficient to define or validate any multi-marker algorithm or to determine an optimal biomarker combination.

From a clinical perspective, these markers can be readily derived from routine preoperative complete blood counts without additional cost. Nevertheless, given that the overall certainty of evidence is low to very low (GRADE), the present results do not support any change in clinical management. Whether concomitantly elevated SIRI and NLR should translate into intensified surveillance, multidisciplinary evaluation, or guideline-concordant adjuvant systemic therapy has not been established by these data and would need to be tested in prospective studies rather than acted upon on the basis of this meta-analysis. Accordingly, the framework integrating SIRI and NLR in **Figure 4** is entirely conceptual and hypothesis-generating, is provided for illustration only, and is not intended to guide current clinical practice or to serve as a risk-stratification algorithm.

Importantly, these biomarkers should be regarded as adjuncts rather than replacements for established clinicopathological factors. Whether their integration with tumour stage, grade, and lymph node status could improve risk stratification remains an open question that would need to be evaluated prospectively (1).

### Implementation considerations

Before clinical implementation, several issues require resolution. First, cut-off values for inflammatory biomarkers varied substantially across studies, limiting direct clinical applicability. Second, few studies assessed whether these biomarkers provide incremental predictive value beyond established clinicopathological models. Third, prospective studies are needed to determine whether biomarker-guided surveillance or adjuvant treatment strategies can improve patient outcomes. Therefore, the SIRI–NLR framework presented here should be interpreted as a conceptual, hypothesis-generating illustration rather than a clinical decision aid.

### Limitations

This study has several limitations. First, all included studies were retrospective, introducing potential selection bias and resulting in low to very low certainty of evidence according to GRADE. Second, substantial heterogeneity in biomarker cut-off values limits direct clinical applicability. Third, most studies were conducted in Asian populations, which may affect generalisability to Western cohorts. Fourth, and most importantly for interpretation, the biomarkers were pooled in separate meta-analyses and were not compared head-to-head within a single statistical model; because the contributing studies also differed in populations and cut-off values, all between-biomarker comparisons—including the apparent ordering of effect sizes and the proposed SIRI–NLR framework—are descriptive and exploratory only. The number of studies evaluating SIRI was also small (three for OS and two for CSS), so no conclusion about its relative superiority can be drawn; for the same reason, SIRI and PLR could not be pooled for RFS. Fifth, because every biomarker–endpoint combination included fewer than 10 studies, formal assessment of publication bias by funnel-plot asymmetry or Egger’s test was not reliable and was therefore not performed; the possibility of small-study effects cannot be excluded. Sixth, study quality was assessed using the Newcastle-Ottawa Scale rather than a tool designed specifically for prognostic-factor studies; although the NOS is widely used for observational prognostic meta-analyses, we acknowledge that the Quality In Prognosis Studies (QUIPS) tool is increasingly recommended for this purpose, and the use of the NOS rather than QUIPS is a methodological limitation of the present review. Seventh, because only aggregated study-level data were available, we could not assess the independent influence of tumour stage, perioperative systemic therapy, biomarker assay timing, or other patient-level confounders on the prognostic effect of each biomarker, nor whether the biomarkers add value beyond established clinicopathological models. Eighth, this review focussed on preoperative inflammatory biomarkers, and postoperative or dynamic changes in inflammatory indices were beyond the scope of the present analysis (26).

### Future research directions

Future studies should focus on multicentre, prospective cohorts that include racially and ethnically diverse populations to externally validate these inflammatory markers, establish standardised cut-off values, and develop integrated risk stratification models that combine systemic inflammation indices with established clinicopathological variables (26). Ultimately, international guidelines from major urological societies will be needed to standardise the incorporation of these biomarkers into routine clinical practice for patients with UTUC.

## Conclusion

Preoperative systemic inflammatory biomarkers are significantly associated with survival outcomes in patients with UTUC undergoing RNU. Each marker was pooled in a separate meta-analysis; because the contributing studies differed substantially in design, populations, and cut-off values, the markers were not compared statistically and no conclusion regarding their relative prognostic performance can be drawn. Given that the overall certainty of evidence is low to very low, these findings should be regarded as descriptive, exploratory, and hypothesis-generating. They indicate that individual inflammatory markers are associated with prognosis, but they do not establish predictive performance and do not support any change in perioperative risk stratification beyond conventional clinicopathological parameters. Establishing whether these markers—individually or in combination—have clinical value would require prospective, individual-patient-data studies that formally assess discrimination, calibration, and clinical utility, with standardised cut-off values and external validation, before any clinical adoption.

## Data Availability

The original contributions presented in the study are included in the article/**Supplementary Material**. Further inquiries can be directed to the corresponding authors.
