# Supplementary material for: Prognostic performance of preoperative systemic inflammatory biomarkers in upper tract urothelial carcinoma after radical nephroureterectomy: a systematic review and meta-analysis of NLR, MLR, PLR, SII and SIRI
Source: Front Oncol. 2026 Jul 29;16:1899751. doi: 10.3389/fonc.2026.1899751 (PMC13461713; doi:10.3389/fonc.2026.1899751)
Supplement: Supplementary file 1 [file DataSheet1.docx]

**Supplementary Material**

***Prognostic performance of preoperative systemic inflammatory biomarkers in upper tract urothelial carcinoma after radical nephroureterectomy: a systematic review and meta-analysis of NLR, MLR, PLR, SII and SIRI***

Guopeng Wang, Xiang Dong, Xintong Zhang, Chengpeng Gu, Ting Hu, Fei Yang, Dong Wang, Yu Mao, Shangqing Ren

*This file contains Supplementary Tables 1–6 and Supplementary Figures 1–5.*

**Supplementary Table 1 |** Detailed search strategies for each database (last searched: 31 March 2026).

| **Database** | **Search strategy (exact string used)** | **Records retrieved** |
| --- | --- | --- |
| **PubMed** | ("upper tract urothelial carcinoma"[MeSH Terms] OR "upper urinary tract urothelial carcinoma"[Title/Abstract] OR "UTUC"[Title/Abstract] OR "upper tract urothelial cancer"[Title/Abstract]) AND ("neutrophil to lymphocyte ratio"[Title/Abstract] OR "NLR"[Title/Abstract] OR "platelet to lymphocyte ratio"[Title/Abstract] OR "PLR"[Title/Abstract] OR "monocyte to lymphocyte ratio"[Title/Abstract] OR "MLR"[Title/Abstract] OR "systemic immune-inflammation index"[Title/Abstract] OR "SII"[Title/Abstract] OR "systemic inflammation response index"[Title/Abstract] OR "SIRI"[Title/Abstract]) AND ("prognosis"[MeSH Terms] OR "survival"[MeSH Terms] OR "prognostic"[Title/Abstract] OR "outcome"[Title/Abstract] OR "mortality"[Title/Abstract] OR "recurrence"[Title/Abstract]) | 69 |
| **Embase** | ('upper tract urothelial carcinoma'/exp OR 'upper urinary tract urothelial carcinoma':ab,ti OR 'UTUC':ab,ti) AND ('neutrophil to lymphocyte ratio':ab,ti OR 'NLR':ab,ti OR 'platelet to lymphocyte ratio':ab,ti OR 'PLR':ab,ti OR 'monocyte to lymphocyte ratio':ab,ti OR 'MLR':ab,ti OR 'systemic immune-inflammation index':ab,ti OR 'SII':ab,ti OR 'systemic inflammation response index':ab,ti OR 'SIRI':ab,ti) AND ('prognosis'/exp OR 'survival'/exp OR prognostic:ab,ti OR outcome:ab,ti OR mortality:ab,ti OR recurrence:ab,ti) | 380 |
| **Web of Science** | TS=("upper tract urothelial carcinoma" OR "upper urinary tract urothelial carcinoma" OR UTUC) AND TS=("neutrophil to lymphocyte ratio" OR NLR OR "platelet to lymphocyte ratio" OR PLR OR "monocyte to lymphocyte ratio" OR MLR OR "systemic immune-inflammation index" OR SII OR "systemic inflammation response index" OR SIRI) AND TS=(prognosis OR survival OR prognostic OR outcome OR mortality OR recurrence) | 80 |
| **Cochrane Library** | ("upper tract urothelial carcinoma" OR "upper urinary tract urothelial carcinoma" OR UTUC) AND ("neutrophil to lymphocyte ratio" OR NLR OR "platelet to lymphocyte ratio" OR PLR OR "monocyte to lymphocyte ratio" OR MLR OR "systemic immune-inflammation index" OR SII OR "systemic inflammation response index" OR SIRI) AND (prognosis OR survival OR prognostic OR outcome OR mortality OR recurrence) | 3 |

*Total records retrieved from all databases before deduplication: 532.*

**Supplementary Table 2 |** Two-by-two contingency table for inter-reviewer agreement on title/abstract screening (Cohen’s κ = 0.92).

| **Reviewer 1 \ Reviewer 2** | **Include** | **Exclude** | **Total** |
| --- | --- | --- | --- |
| Include | 91 (a) | 7 (b) | 98 |
| Exclude | 5 (c) | 274 (d) | 279 |
| Total | 96 | 281 | 377 |

*Total records screened at the title/abstract stage = 377. a = both reviewers agreed to include; b = Reviewer 1 included, Reviewer 2 excluded; c = Reviewer 1 excluded, Reviewer 2 included; d = both reviewers agreed to exclude. Cohen’s κ = 0.92.*

**Supplementary Table 3 |** GRADE evidence profile for each biomarker–endpoint combination.

| **Endpoint** | **Biomarker** | **k** | **Risk of bias** | **Inconsistency** | **Indirectness** | **Imprecision** | **Certainty** |
| --- | --- | --- | --- | --- | --- | --- | --- |
| CSS | NLR | 4 | Serious (retrospective) | Not serious | Serious (variable cut-offs) | Not serious | Low |
| CSS | PLR | 3 | Serious (retrospective) | Not serious | Serious (variable cut-offs) | Not serious | Low |
| CSS | SII | 4 | Serious (retrospective) | Serious | Serious (variable cut-offs) | Not serious | Very low |
| CSS | SIRI | 2 | Serious (retrospective) | Not serious | Serious (variable cut-offs) | Serious | Very low |
| OS | MLR | 2 | Serious (retrospective) | Not serious | Serious (variable cut-offs) | Serious | Very low |
| OS | NLR | 3 | Serious (retrospective) | Not serious | Serious (variable cut-offs) | Not serious | Low |
| OS | PLR | 3 | Serious (retrospective) | Not serious | Serious (variable cut-offs) | Not serious | Low |
| OS | SII | 4 | Serious (retrospective) | Serious | Serious (variable cut-offs) | Not serious | Very low |
| OS | SIRI | 3 | Serious (retrospective) | Serious | Serious (variable cut-offs) | Not serious | Very low |
| RFS | NLR | 3 | Serious (retrospective) | Not serious | Serious (variable cut-offs) | Not serious | Low |
| RFS | SII | 2 | Serious (retrospective) | Not serious | Serious (variable cut-offs) | Serious | Very low |

*k, number of pooled studies; CSS, cancer-specific survival; OS, overall survival; RFS, recurrence-free survival. All bodies of evidence started as observational (low) and were further downgraded; none was upgraded. Certainty rated as low to very low overall.*

**Supplementary Table 4 |** Summary of biomarker cut-off values reported in the included studies.

| **Study** | **Year** | **Biomarker** | **Cut-off (high vs low)** |
| --- | --- | --- | --- |
| Boubaker_2025 | 2025 | MLR | ≥0.358 vs <0.358 |
| Boubaker_2025 | 2025 | NLR | ≥1.5 vs <1.5 |
| Boubaker_2025 | 2025 | PLR | ≥175 vs <175 |
| Boubaker_2025 | 2025 | SII | ≥668 vs <668 |
| Boubaker_2025 | 2025 | SIRI | >1.904 vs <1.904 |
| Chien_2021 | 2021 | NLR | ≥3.5 vs <3.5 |
| Chien_2021 | 2021 | NLR | ≥2.9 vs <2.9 |
| Chien_2021 | 2021 | PLR | ≥162 vs <162 |
| Chien_2021 | 2021 | PLR | ≥150 vs <150 |
| Chien_2021 | 2021 | SII | ≥460 vs <460 |
| Chien_2021 | 2021 | SII | ≥485 vs <485 |
| Chien_2021 | 2021 | SII | ≥490 vs <490 |
| Dalpiaz_2017 | 2017 | PLR | ≥150 vs <150 |
| Ghorai_2024 | 2024 | NLR | ≥2.5 vs <2.5 |
| Jan_2019 | 2019 | MLR | ≥0.4 vs <0.4 |
| Jan_2019 | 2019 | SII | ≥580 vs <580 |
| Tanaka_2014 | 2014 | NLR | ≥3.0 vs <3.0 |
| Mori_2021 | 2021 | SII | ≥485 vs <485 |
| Son_2018 | 2018 | NLR | >3.3 vs ≤3.3 |
| Son_2018 | 2018 | PLR | >142 vs ≤142 |
| Tan_2018 | 2018 | NLR | ≥2.5 vs <2.5 |
| Yu_2024 | 2024 | SIRI | ≥1.95 vs <1.95 |
| Zheng_2019 | 2019 | PLR | ≥126.88 vs <126.88 |
| Zheng_2019 | 2019 | SIRI | ≥1.36 vs <1.36 |
| Zheng_2020 | 2020 | SII | ≥672.44 vs <672.44 |

**Supplementary Table 5 |** Leave-one-out sensitivity analysis for the systemic immune-inflammation index (SII) in overall survival.

| **Study omitted** | **Pooled HR (95% CI)** | **I² (%)** |
| --- | --- | --- |
| Omitting Boubaker_2025 | 1.53 (1.00–2.34) | 58.9 |
| Omitting Jan_2019 | 1.47 (1.08–2.01) | 88.9 |
| Omitting Mori_2021 | 1.70 (1.55–1.87) | 0 |
| Omitting Zheng_2020 | 1.51 (1.10–2.08) | 89.4 |

*Omission of Mori et al. (2021) reduced heterogeneity from I² = 84.5% (full model) to 0%, identifying it as the principal source of heterogeneity. HR, hazard ratio; CI, confidence interval.*

**Supplementary Table 6 |** Sensitivity analysis for overall survival with the neutrophil-to-lymphocyte ratio (NLR), excluding the statistical outlier study.

| **Model** | **k** | **Pooled HR (95% CI)** | **I² (%)** |
| --- | --- | --- | --- |
| All studies | 3 | 1.97 (1.74–2.22) | 14.1 |
| Excluding Ghorai et al. (2024) | 2 | 1.97 (1.86–2.08) | 0 |

*Excluding the outlier (Ghorai et al., 2024; reported HR ≈ 9.88) left the pooled estimate essentially unchanged while removing all heterogeneity. k, number of pooled studies.*

**Supplementary Figure 1 |** Summary of the methodological quality of included studies assessed using the Newcastle-Ottawa Scale (NOS).

**Supplementary Figure 2 |** Forest plots of all separate random-effects meta-analyses for the five inflammatory biomarkers across overall survival (OS), cancer-specific survival (CSS), and recurrence-free survival (RFS). (Panels continue over the following pages.)

**Supplementary Figure 3 |** Subgroup analyses of the systemic inflammation response index (SIRI) for overall survival, stratified by geographic region, sample size, and NOS score.

**Supplementary Figure 4 |** Leave-one-out sensitivity analysis for the SII–overall survival meta-analysis.

**Supplementary Figure 5 |** Baujat plot identifying the contribution of each study to the heterogeneity of the SII–overall survival meta-analysis.
