# Supplementary material for: Prognostic performance of preoperative systemic inflammatory biomarkers in upper tract urothelial carcinoma after radical nephroureterectomy: a systematic review and meta-analysis of NLR, MLR, PLR, SII and SIRI
Source: Front Oncol. 2026 Jul 29;16:1899751. doi: 10.3389/fonc.2026.1899751 (PMC13461713; doi:10.3389/fonc.2026.1899751)
Supplement: Supplementary file 2 [file Table1.docx]

**PRISMA 2020 Checklist**

***Prognostic performance of preoperative systemic inflammatory biomarkers in upper tract urothelial carcinoma after radical nephroureterectomy: a systematic review and meta-analysis of NLR, MLR, PLR, SII and SIRI***

*Completed in accordance with the PRISMA 2020 statement (Page MJ, et al. BMJ. 2021;372:n71). Section names refer to the submitted manuscript; please add page or line numbers as required by the journal.*

| **Section and topic** | **Item** | **Checklist item** | **Location where item is reported** |
| --- | --- | --- | --- |
| **TITLE** | | | |
| Title | 1 | Identify the report as a systematic review. | Title |
| **ABSTRACT** | | | |
| Abstract | 2 | See the PRISMA 2020 for Abstracts checklist (structured summary). | Abstract |
| **INTRODUCTION** | | | |
| Rationale | 3 | Describe the rationale for the review in the context of existing knowledge. | Introduction |
| Objectives | 4 | Provide an explicit statement of the objective(s) or question(s) the review addresses. | Introduction; Methods (PECO framework) |
| **METHODS** | | | |
| Eligibility criteria | 5 | Specify inclusion/exclusion criteria and how studies were grouped for syntheses. | Methods — Inclusion and exclusion criteria |
| Information sources | 6 | Specify all databases/registers searched and the date each was last searched. | Methods — Search strategy and study selection |
| Search strategy | 7 | Present the full search strategies for all sources. | Methods; Supplementary Table 1 |
| Selection process | 8 | Specify methods to decide whether a study met inclusion criteria (independent reviewers). | Methods — Search strategy and study selection; Supplementary Table 2 |
| Data collection process | 9 | Specify methods used to collect data from reports (independent reviewers). | Methods — Data extraction and quality assessment |
| Data items | 10a | List and define all outcomes for which data were sought. | Methods — Statistical analysis; Inclusion criteria |
| Data items | 10b | List and define all other variables for which data were sought. | Methods — Data extraction; Table 1; Supplementary Table 4 |
| Study risk of bias assessment | 11 | Specify the tool(s) used to assess risk of bias and the process (Newcastle-Ottawa Scale). | Methods — Data extraction and quality assessment; Supplementary Figure 1 |
| Effect measures | 12 | Specify the effect measure(s) used in the synthesis (hazard ratio with 95% CI). | Methods — Statistical analysis |
| Synthesis methods | 13a | Describe processes used to decide which studies were eligible for each synthesis. | Methods — Statistical analysis; Results — Meta-analysis |
| Synthesis methods | 13b | Describe methods to prepare data for synthesis (log HR and standard error; inverse-variance). | Methods — Statistical analysis |
| Synthesis methods | 13c | Describe methods used to tabulate or visually display results. | Results; Figures 2–3; Supplementary Figure 2 |
| Synthesis methods | 13d | Describe methods to synthesise results (random-effects model, DerSimonian-Laird; I²). | Methods — Statistical analysis |
| Synthesis methods | 13e | Describe methods used to explore heterogeneity (subgroup analyses). | Methods — Statistical analysis; Results — Subgroup and sensitivity analyses |
| Synthesis methods | 13f | Describe any sensitivity analyses. | Methods — Statistical analysis; Results; Supplementary Tables 5–6 |
| Reporting bias assessment | 14 | Describe methods to assess risk of bias due to missing results. | Methods — Statistical analysis (not performed; <10 studies per analysis); Discussion — Limitations |
| Certainty assessment | 15 | Describe methods used to assess certainty in the body of evidence (GRADE). | Methods — Statistical analysis; Supplementary Table 3 |
| **RESULTS** | | | |
| Study selection | 16a | Report numbers screened and included, ideally with a flow diagram. | Results — Study selection; Figure 1 |
| Study selection | 16b | Cite studies excluded at full-text stage and give reasons. | Figure 1 (PRISMA 2020 flow diagram) |
| Study characteristics | 17 | Cite each included study and present its characteristics. | Results — Study characteristics; Table 1 |
| Risk of bias in studies | 18 | Present risk-of-bias assessments for each included study. | Table 1; Supplementary Figure 1 |
| Results of individual studies | 19 | For each study, present effect estimates and precision. | Supplementary Figure 2; Table 1 |
| Results of syntheses | 20a | For each synthesis, summarise characteristics and risk of bias of contributing studies. | Results — Meta-analysis |
| Results of syntheses | 20b | Present results of each synthesis: pooled estimate, CI, and heterogeneity. | Results — Meta-analysis; Figure 2; Table 2 (pooled estimates) |
| Results of syntheses | 20c | Present results of investigations of possible causes of heterogeneity. | Results — Subgroup and sensitivity analyses; Supplementary Figure 3 |
| Results of syntheses | 20d | Present results of all sensitivity analyses. | Results — Subgroup and sensitivity analyses; Supplementary Tables 5–6; Supplementary Figures 4–5 |
| Reporting biases | 21 | Present assessments of risk of bias due to missing results for each synthesis. | Discussion — Limitations |
| Certainty of evidence | 22 | Present assessments of certainty in the body of evidence for each outcome. | Results; Discussion; Supplementary Table 3 |
| **DISCUSSION** | | | |
| Discussion | 23a | Provide a general interpretation of the results in the context of other evidence. | Discussion |
| Discussion | 23b | Discuss any limitations of the evidence included in the review. | Discussion — Limitations |
| Discussion | 23c | Discuss any limitations of the review processes used. | Discussion — Limitations |
| Discussion | 23d | Discuss implications for practice, policy, and future research. | Discussion — Clinical implications; Future research directions; Conclusion |
| **OTHER INFORMATION** | | | |
| Registration and protocol | 24a | Provide registration information, including register name and registration number. | Methods — Search strategy and study selection (PROSPERO CRD420261307425) |
| Registration and protocol | 24b | Indicate where the review protocol can be accessed. | PROSPERO (https://www.crd.york.ac.uk/PROSPERO/view/CRD420261307425) |
| Registration and protocol | 24c | Describe and explain any amendments to information provided at registration. | Amendments to the registered protocol, made before data analysis and consistent with the PROSPERO revision history: (1) the pre-registered network meta-analysis was replaced by separate random-effects meta-analyses for each biomarker owing to an insufficiently connected evidence network; (2) the systemic inflammation response index (SIRI) replaced the fibrinogen-to-albumin ratio (FAR) as the fifth biomarker, as FAR was reported by fewer than three studies; (3) risk of bias was assessed with the Newcastle-Ottawa Scale rather than the pre-registered Cochrane risk-of-bias tool, because all included studies were retrospective observational cohorts; (4) the pre-specified publication-bias assessment was not performed because every biomarker–endpoint combination included fewer than 10 studies. Reported in Methods (Search strategy and study selection; Statistical analysis). |
| Support | 25 | Describe sources of financial or non-financial support and the role of funders. | Funding |
| Competing interests | 26 | Declare any competing interests of review authors. | Conflict of interest |
| Availability of data, code and other materials | 27 | Report which of the following are publicly available and where: data, analysis code, other materials. | Data availability statement; analysis code (R script) available from the corresponding authors on request |

*Abbreviations: CI, confidence interval; CSS, cancer-specific survival; GRADE, Grading of Recommendations Assessment, Development and Evaluation; HR, hazard ratio; OS, overall survival; PECO, Population-Exposure-Comparator-Outcome; RFS, recurrence-free survival.*
